# Notes on a Fatal Case of Quiescent Gall Stones

**Published:** 1831

**Authors:** Daniel Drake

**Affiliations:** Cincinnati


### Art. IV.—*Notes of a Fatal Case of Quiescent Gall Stones.*—By Daniel Drake, M. D.

The subject of this report was M. L., a gentleman 76 years of age. His life, for the first half, was one of activity and hardship—great part of it being passed on the ocean: for the remaining portion, his pursuits were sedentary. For most of the former period, he was accustomed to the daily use of ardent spirits; during the latter, up to the time of his death, he discontinued their use, and did not even substitute wine. His diet was generally temperate and simple; and still, for 30 or 40 years of the latter part of his life, he was corpulent; and did not begin to emaciate till two or three years before his death.

At the age of 40, or thereabouts, he became afflicted with gout, which continued to return upon him every winter, till within two or three years of his death. It was often severe, affecting his toes, knees, and head. Not far from the same period, he was seized with diarrhœa, which afflicted him, through the whole of that long period, to the time of his death. He was subject, for several years, to extraordinary paroxysms of gastric flatulence and eructation, which were more certainly relieved by a draught of warm water, than any other remedy. He never vomited, till near the close of life. He was not subject to colic; nor ever experienced an attack of jaundice. He once had an attack of ague and fever, when comparatively a young man. In all the latter years of his life, the diarrhœa constituted his greatest affliction. It was but little under the control of medicines, or dietetic methods of any kind. Eighteen months or two years before his death, he began to lose flesh, and his emaciation continued to increase up to the day of his death, when it was extreme, especially in the organs of the abdomen. The contrast between the extended basis of the thorax, and the size of the abdomen was the most striking I have ever seen. For some weeks before his death, the diarrhœa was so incessant, that he would rise 40 or 50 times in the 24 hours. The discharges were generally composed of yellowish water, with great volumes of gas. For two or three weeks before his death he had perpetual nausea, and sometimes, vomited bile. He also complained of an incessantly bitter and most offensive taste. His tongue was slightly furred. His pulse was, at no time, for the last few weeks of his life, in a state of excitement; but, the reverse—weak and slow. He could lie on his back, and on either side. His abdomen was carefully examined, but nothing appeared, except extreme atrophy of the organs.

I was unable to form a satisfactory opinion as to the kind of irritation which kept up the peritaltic action. The liver evidently secreted and threw out bile, the mesenteric glands were not enlarged, and he did not discharge pus from the bowels. My efforts to arrest the diarrhœa, were ineffectual, and the patient desired a consultation. Drs. Marshal and Staughton were called in. They were equally at a loss with myself. We pursued a routine and empirical practice—giving, in succession or combination, minute and even considerable doses of the pil. Hydrargyri and Calomel—small doses of Ipecacuanha—the Cretacevus Mixture—Acetate of Lead—Tincture of Kino—the Sulphate of Quina—decoction of Peruvian Bark—Sulphate of Morphia—Laudanum, and solid Opium—with external irritation to the epigastrium, and a liquid diet, of which fecula and gluten were the basis. An emetic was also administered, and two or three times he took an active cathartic. But the whole proved entirely unavailing. The diarrhœa continued to increase, and his flesh and strength to diminish, until, at length, on the 13th of July, 1831, he expired.—For many years before death, his temper was gloomy, discontented, and unhappy.

Appearances on Dissection.—Leave having been freely granted, to examine the abdominal and thoracic viscera, it was done on the next day after death.

The great degree of emaciation, of which I have spoken, was more particularly evident in the abdomen, and yet, a considerable stratum of adipose matter, was found between the skin and abdominal muscles. The omentum was extremely attenuated; and the viscera, generally, exhibited a corresponding atrophy. The small intestines had sunk into the pelvis. The mesentery, free from tubercular induration, but presented a dirty, yellowish-green color, which contrasted strongly with the engorged veins that were creeping over its surface. The colon was contracted into a tubular cord, and wore a shrivelled appearance. The spleen was small, but healthy. The pancreas was sound. The mucous membrane of the stomach and intestines throughout, was *entirely free from ulceration*, and displayed no remarkable color, except in the greater curvature of the stomach, which was engorged. They abounded in mucus, and contained a considerable quantity of healthy bile. In several places their parieties were unusually tender, and appeared to have been slightly inflamed. The liver was rather small, but totally free from disease. *The gall-bladder was elongated, and thinned in its parieties. On touching it, the existence of numerous biliary calculi, angular, and greatly indurated, was at once apparent. By shaking the liver, their rattling was so loud, that it is a matter of astonishment, that the patient had not sometimes heard them, when alive and in motion.*

The lungs were sound. The heart was unusually small; but healthy, except a slight deposit of osseous matter, on the edges of the semilunar valves. There were no urinary calculi.

Examination of the Calculi.—This was made by Mr. Shotwell, one of my pupils. The cystic duct was permanently contracted, nearly to obliteration. The cyst contained a little slimy fluid colored with bile. Its inside exhibited but little appearance of a mucous membrane, being nearly smooth.

The calculi were seventy-three in *number*, and varied in size, from that of a pea, to that of a hazle nut.

The *form* of nearly the whole, was a tetrahedron, or solid triangle, a figure which a few of them affected with surprising accuracy; while two or three approached nearly to the cube. In all cases, the angles rounded.

Their *surface* was polished, and soapy to the touch.

The *color* bore a resemblance to some varieties of serpentine—consisting in greenish-yellow clouds, inserted into a dark-brown field.

Such as were examined internally, exhibited a concentric, lamellar *structure*, except the centre, which was homogeneous, irregular, and nearly black.

They were *fractured* by a gentle blow, and could be easily acted on by the nail.

Their *specific gravity* was such, that they all sunk in alcohol; but most of them swam in water. The few that sunk, after some time, became surrounded with a multitude of minute, adherent air bubbles, and rose to the surface.

They were *insoluble* in water, alcohol, or oil of turpentine. In the flame of a candle, they *burnt* like sealing wax.

In *taste*, they were insipid.

In the *Dictionaire des Sciences Medicales*, Art. Calculs Biliaires, we have the following classification of Gall Stones:

" The biliary calculi, hitherto examined, may be divided into four classes: 1st. Those which present a white color, are crystalline, brilliant, lamellar, and composed entirely of adipocire. 2d. Those which are composed in part of that substance, and in part of a brown substance, which appears to be nothing else than inspissated bile: they are polygonal, and their color is a greyish brown. 3d. Those which seem to be formed entirely of concrete bile. 4th. and lastly, non-combustible calculi, but which are nevertheless consumed little by little, when exposed to an intense heat.

The gall stone of the first class, has generally an ovoid shape; its volume never exceeds that of a pigeon's egg, while its common size is that of a sparrow's; however, Saye found one in the gall bladder of a woman, nineteen years old, which was equal to a hen's egg.—(*Jour. des Sav., Sept.* 1697.) The cyst rarely contains more than one of this species; nevertheless, Dr. Merat has mentioned in his memoir, on Adipocire, (*Soc. Med. d'Emul.*) the case of a woman in whose gall bladder he found eighty-three.

The calculi of this class have a white color, a little yellow on the exterior: their interior structure is lamellar, crystalline, and in shining plates or striæ: their weight is less than that of water: exposed to a high temperature, they soften and melt: they are insoluble in water: hot alcohol dissolves them, but on cooling, the matter is deposited in brilliant laminæ: they are equally soluble in spirits of turpentine, and the caustic alkalies,—in the last case the solution having all the properties of soap.

Calculi of the second class, are always found in great numbers, a circumstance that necessarily affects their form, which is generally triangular, with the edges and points rounded; their specific gravity is very variable. [*Thomson and Bostock.*] The former has analyzed many, which constantly sunk in water: their exterior surface is smooth and soft to the touch; they are formed of concentric laminæ, arranged with small rays inclining towards the centre. The composition of this species, differs from the last, in this only, that they always contain a brownish ingredient, which possesses most of the characters of the resin of the bile.

The gall stones of the third class, are most frequently found in animals, particularly the ox, but sometimes in man.—(*Thenard. Mem. d'Arcueil.*) They are composed of inspissated bile: their figure is irregular; they have a bitter taste, and sometimes considerable hardness.

Haller and Saunders, are almost the only persons who have spoken of calculi of the fourth class: they are insoluble in alcohol, and the oil of turpentine. Some of them are uninflammable, but will redden and consume like charcoal."

### Remarks.

1. The calculi taken from the gentleman, whose case is here reported, evidently belong to the second class, of the preceding arrangement.

2. They had probably been formed soon after he changed his habits, from active to sedentary, more than thirty years before his death. What connexion their formation might have had with his arthritic diatheses, I am not prepared to say.

3. It is worthy of remark, that they occasioned no disease of the liver, nor even one attack of jaundice.

4. The sympathetic affections which they excited, were paroxysms of gastrodynia and flatulence, chronic diarrhœa, depression of spirits, and, ultimately, great emaciation.

5. It is probable, that many cases of chronic diarrhœa, which resist all kinds of remedies, and are supposed to depend on ulceration of the mucous membrane of the bowels, are referable to this cause; and may not many cases of hypochondriacism be occasioned in the same manner?

6. And lastly, the effect, on the vital powers, of the sojourn of these concretions in the gall bladder, seems to have been, at all times, directly enervating. For many years, this influence was inconsiderable; but it finally led to great debility in the circulation, with an atrophy which extended to the heart, as well as to other organs.

*Cincinnati, Sept.*, 1831.

### DR. DRAKE'S RESIGNATION.

*To the Honorable Board of Trustees of the Medical College of Ohio.*

GENTLEMEN,

Last summer, when I suggested and agreed to accept the chair of Clinical Medicine, in the Medical College of Ohio, as the only means in my power, of effecting the union of the Medical Department of Miami University, with the College, an object which you had originated and were anxious to accomplish, my chief motive was to prevent the interests of medical education in Cincinnati, from being seriously injured by a protracted negotiation. At the moment of devising the professorship, I felt an apprehension, that its subjects and limits would be too restricted, in the existing condition of the Hospital, to afford me an opportunity of being useful to the class; but I felt and perceived, that a state of things had been produced by others, which left me no alternative, and I accepted the chair, resolving, if my apprehensions should be realized, to resign it at the end of the first session.

When the chair of the Institutes was declined by the gentleman, (*Dr. Pierson of New York*) to whom it was offered, unless he were allowed a year for preparation, and I saw that branch assigned to me, (*for the time being*,) by the unanimous vote of the Faculty, I cherished the hope, that your honorable body would not grant the favor which he solicited; but, at once, confirm the union of the Institutes with Clinical Medicine, and by reducing the number of chairs from eight to seven, afford me a sufficient amount of duties. You have, however, preferred a different course, and I am, therefore, compelled to request you to accept my resignation, which I wish to take place immediately after the next Commencement.

Thirteen years have this day elapsed, since I had the honor to solicit and obtain from our Legislature, the charter of the College; and to be appointed President and Professor of the Institutes and Practice of Medicine. From that day down to the present anniversary, whether belonging to it or to other institutions, in different and distant cities, the Medical College of Ohio has never ceased, nor can it hereafter cease, to be with me an object of the deepest interest. Therefore it is, that I send in my resignation before the end of the session, that your honorable body may be able to make and promulgate arrangements for the next course of lectures, before the class has dispersed.

In taking a final leave of an institution, which for so many years has been an object of affection, but to the prosperity of which I can no longer contribute the influence of my feeble exertions, I cannot forego the opportunity of expressing a hope, that it will continue to flourish until it shall reach the high distinction and impart the solid advantages, anticipated in its foundation.

I have the honor to be respectfully,

Your ob't serv't,

DANIEL DRAKE.

*Cincinnati, Ohio, Jan.* 19, 1832.

